# CO_2_ Reduction by Nanosecond-Plasma Discharges: Revealing the Dissociation’s Time Scale and the Importance of Pulse Sequence

**DOI:** 10.1021/acs.jpcc.3c02547

**Published:** 2023-05-18

**Authors:** Cesare Montesano, Toine P.W. Salden, Luca Matteo Martini, Giorgio Dilecce, Paolo Tosi

**Affiliations:** † Department of Physics, University of Trento, Trento, 38123, Italy; ‡ Department of Applied Physics, Eindhoven University of Technology, Eindhoven, 5600MB, Netherlands; ¶ CNR Institute for Plasma Science and Technology, Bari, 70126, Italy

## Abstract

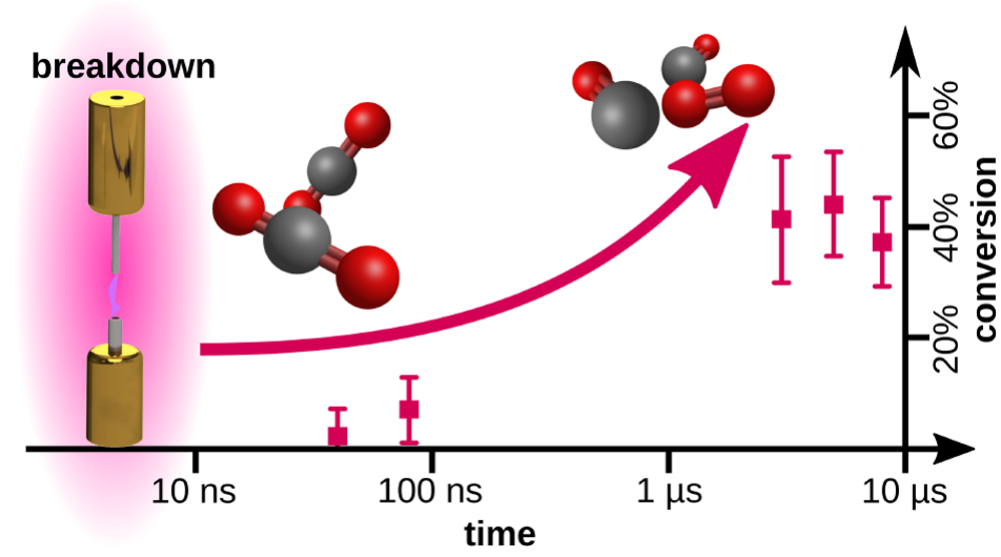

Power-to-chemical technologies with CO_2_ as feedstock recycle CO_2_ and store energy into value-added compounds. Plasma discharges fed by renewable electricity are a promising approach to CO_2_ conversion. However, controlling the mechanisms of plasma dissociation is crucial to improving the efficiency of the technology. We have investigated pulsed nanosecond discharges, showing that while most of the energy is deposited in the breakdown phase, CO_2_ dissociation only occurs after an order of microsecond delay, leaving the system in a quasi-metastable condition in the intervening time. These findings indicate the presence of delayed dissociation mechanisms mediated by CO_2_ excited states rather than direct electron impact. This “metastable” condition, favorable for an efficient CO_2_ dissociation, can be prolonged by depositing more energy in the form of additional pulses and critically depends on a sufficiently short interpulse time.

## Introduction

The daunting task of reducing the emission of greenhouse gases in the atmosphere and finding alternative energy sources to fossil fuels burdens our societies. The late Nobel prize laureate G. Olah proposed to produce synthetic fuels using CO_2_ and green electricity to close the anthropogenic carbon cycle;^1^ at the same time, renewable energy is stored in value-added compounds.^2,3^ For this purpose, plasma-catalysis-based technology^4−11^ is among the different implementations under development.^12−16^ In particular, atmospheric nanosecond repetitively pulsed (NRP) discharges have shown high efficiencies in converting CO_2_,^17−20^ providing strong motivation for fundamental investigations.^21,22^ The core idea behind the plasma approach is exploiting the nonequilibrium features of gaseous discharges, which ideally allow directing the energy into CO_2_ dissociation with minimum gas heating.

Despite increasing research, CO_2_ conversion via plasma has yet to reach the maturity needed for industrial applications. Not surprisingly, the challenge for upgrading plasma technologies to a commercially viable level is the precarious balancing of bulk conversion versus overall energy efficiency. The enthalpy of CO_2_ reduction to CO + O is 5.5 eV molecule^–1^. In turn, atomic oxygen can react with CO_2_, so the reaction enthalpy of
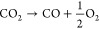
1is 2.9 eV molecule^–1^.

In plasmas, electrons trigger CO_2_ dissociation via excitation to electronic dissociative states or via vibrational excitation. While the first mechanism requires energies substantially higher than the 5.5 eV threshold, dissociation via vibrational excitation can take advantage of vibrational energy transfer collisions that can pump some molecules up to the dissociation limit from the ground state (vibrational ladder climbing mechanism). The interplay of all these electron-initiated processes determines the final energy required for the plasma reduction of CO_2_. Thus, controlling the dissociation mechanisms is the key to obtaining a product yield and energy efficiency suited to viable applications. Reaching this goal requires a novel approach to the experimental investigation of plasma chemistry; local, time-resolved diagnostics are needed to look inside the plasma at the proper time scale beyond the mere quantification of the reaction products leaving the reactor.

In our first attempt,^23^ we used time-resolved laser-induced fluorescence (LIF) to measure the CO_2_ dissociation in the 4–150 μs interval after a nanosecond discharge. The observed time evolution indicates a high dissociation degree a few μs after the discharge that then monotonically decreases to the asymptotic steady-state value observed at the exit of the reactor. An important conclusion was that a further increase of the discharge energy is not critical; instead, back reactions and remixing with untreated gas are likely the limiting factors. However, the evolution of CO_2_ dissociation in the μs-time scale cannot give an insight on the fundamental mechanisms responsible for the dissociation process.

To answer this question, in the present work, we have brought the LIF measurements 2 orders of magnitude (40 ns) closer to the discharge pulse, observing that the dissociation degree gradually increases, with the peak value in the μs-scale. These findings shed light on the dissociation’s time scale and can hint at the prevailing mechanisms. In addition, we have extended measurements to a sequence of successive pulses. We find that the timing with which the plasma is sustained is crucial regarding energy dissipation, CO_2_ dissociation, and gas temperature.

## Methods

Our pulsed nanosecond discharge operated at atmospheric pressure, and the flow of CO_2_ flow was kept constant at 100 sccm (standard cubic centimeter). The discharge geometry reproduced the configurations employed in other works.^19,24^ The electrodes were arranged in a pin-to-pin configuration; the high voltage (HV) electrode was a stainless steel tube, which serves also as the gas inlet. The grounded electrode was a stainless steel tube (3 mm outer diameter) with the additional purpose of the gas outlet. The interelectrode gap was set at 5 mm.

A HV power supply NPG18/100k (Megainpulse Ltd.) was employed to produce the HV pulses, characterized by an full width at half-maximum of about 10 ns and rise time <4 ns on a 75 Ω load. The maximum pulse frequency is 100 kHz, while the maximum number of pulses per second is equal to 4000. The NPG18/100k was externally triggered by a waveform generator (33220A, Agilent Technology).

**Figure 1:**
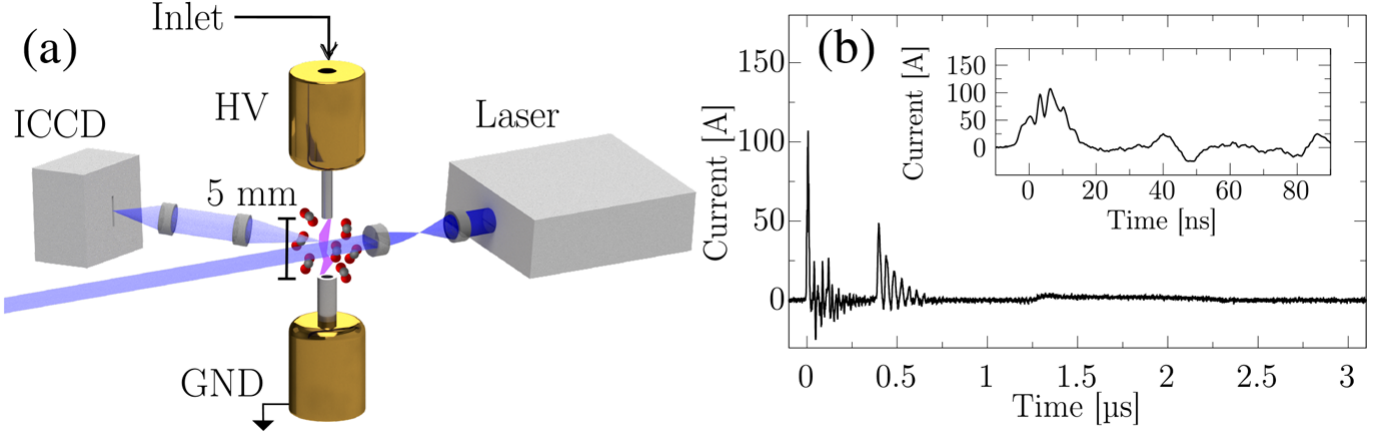
(a) Simplified 3D view of the discharge setup and the light generation, manipulation, and collection. HV: high-voltage electrode; GND: ground electrode. The details on the intensified-CDD, laser radiation, and light manipulation are provided in the SI. (b) Example of a current signal resulting from a high-voltage pulse with full width at half-maximum of 10 ns in the transmission line.

At around 400 ns after the trigger signal, a spurious retrigger event occurred that, in some cases, is able to reignite the discharge; see Figure S12 in the Supporting Information (SI). An I/V converter (CT-D-1.0, Magnelab, 200 Hz to 500 MHz bandwidth) and a high-voltage probe (P6015A Tektronix, 75 MHz bandwidth) were used to measure the current and voltage signals, respectively. A digital oscilloscope (WaveSurfer 104-Mx, 1 GHz bandwidth) acquired and digitized the signals with a sampling rate of 5 GS/s. A digital delay generator (DDG) synchronizes the HV power supply, the light detection, and the laser. The HV pulse was injected into the transmission line ended with the load (the reactor). The residual power traveling back and forth in the cable could occasionally reignite the discharge. A typical current trace is shown in Figure 1b, and details on the I/V characteristics of the ns pulses are reported in the SI.

Adding a trace amount of water allows the generation in the plasma of OH, which is laser excited to the state OH  at the desired time delay to the discharge (see SI). A spectrograph with a 300 mm focal length monochromator (SR303i-B, Shamrock, 2400 and 1200 grooves/mm gratings) and gated intensified-CCD (ICCD, DH334T-18U-03, Andor iStar) camera collects the fluorescence. A simplified sketch of the experimental setup is reported in Figure 1a.

The second harmonic of a 10 Hz Q-switched Nd:YAG laser (YG580, Quantel) was used to pump a frequency-doubled tunable dye laser (TDL50, Quantel; Rhodamine 590 chloride dye). The dye laser was tuned to excite the P_1_(3) line of the
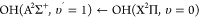
2transition at 283.009 nm. A beam energy of 450 μJ per pulse was employed. The initial circular (4 mm diameter) shape of the laser beam was adapted to obtain a planar geometry (1 × 4 mm) perpendicular to the interelectrode axis by means of a telescope consisting of two plano-convex cylindrical lenses. This beam geometry ensures that the laser always intercepts the discharge channel, which occurs randomly in the space of the gap. The wavelength was fixed by a feedback loop composed of an etalon, a CCD, and a piezoelectric actuator.

Energy-transfer collisions with the background molecules depopulate the laser-excited state nonradiatively and influence the fluorescence spectra. Thus, information on gas composition and CO_2_ dissociation can be retrieved by analyzing the LIF outcomes recorded at various times after the discharge pulse. We called this procedure, which uses OH as a sensor of the background gas composition, collision-energy-transfer laser-induced-fluorescence, CET-LIF.^23,25,26^

CET-LIF analysis requires knowledge of the gas temperature *T* because of the *T*-dependence of the energy-transfer rate coefficients.^25^ For this purpose, we added 5% of nitrogen to the CO_2_ stream and recorded the emission of nitrogen’s second positive system (SPS):
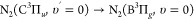
3to estimate the gas (translational) temperature.^27,28^ In atmospheric pressure discharges, the equivalence between the rotational temperature *T*_rot_ of the emitting state and the gas temperature is generally wrong since the emitting state cannot usually reach the thermal equilibrium during its lifetime. However, if the rotational population distribution of the emitting state replicates that of the ground state, the temperature determined by the optical emission matches the gas temperature. The latter condition is fulfilled if the  is produced by electron impact, while other mechanisms that can influence the population distribution (e.g., pooling or chemical reactions) can be safely neglected. This condition is fulfilled in the present work because the emission of the SPS is collected in the first nanoseconds of the discharge.

To record the time evolution of *T*, the  state is populated at different delays in the postdischarge by an auxiliary HV pulse. For technical reasons, the additional pulse could not be closer than ten μs, which limited the minimum delay after the discharge (below 10 μs the gas temperature was extrapolated using a double exponential fit as in ref (24), see paragraph 3 of the SI). However, at 40 and 80 ns, we could exploit secondary discharges caused by reflections in the transmission line connecting the HV power supply to the reactor due to impedance mismatch. At later times, the reflected power is too low to ignite a further discharge.

Provided that the CO_2_ dissociation develops in accordance with the reaction in eq 1, the gas mixture composition after a given delay from the discharge can be parametrized with a single parameter γ:
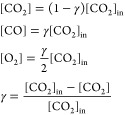
4where  represent the initial CO_2_ concentration. The CO_2_ dissociation *C* [%] is defined as
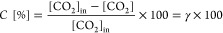
5

For further details on experimental design and operation, see the SI.

## Results and Discussion

**Figure 2:**
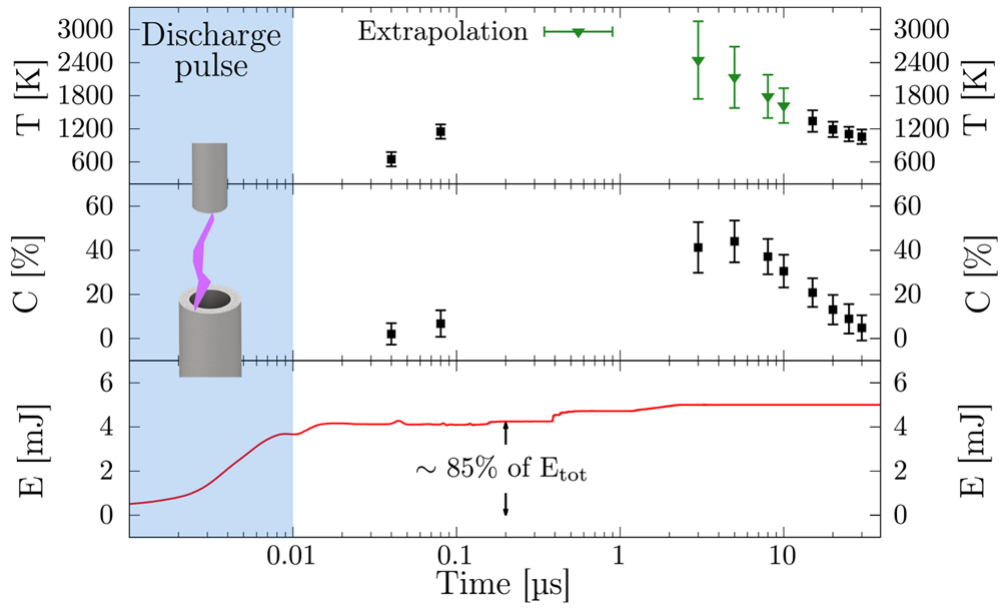
Top to bottom panels: gas temperature, CO_2_ conversion, and energy during the first pulse of a burst. The green markers in the gas temperature panel indicate the extrapolated values; see the SI. The 3D view of the electrodes in the blue shaded area represents the discharge event happening in the first 10 ns.

Figure 2 shows the pulse’s energy *E* [mJ], the gas temperature *T* [K], and the CO_2_ dissociation *C* [%] as a function of the time delay to the discharge. A striking observation concerns the low CO_2_ reduction to CO and O_2_ (<8%) up to 80 ns after the breakdown, although about 85% of the discharge energy has already been dissipated. After 5 μs, the CO_2_ dissociation is about 45%.

The molecular dissociation appears to rise progressively after the breakdown, suggesting that the direct excitation to a dissociative state by electron impact is not the leading cause. Instead, the delay between electron impact and dissociation hints at an indirect mechanism mediated by CO_2_ excitation. This interpretation agrees with the results provided by time-resolved optical emission spectroscopy (OES).^24^ There, two different regimes of the discharge evolution were reported. An initial breakdown phase, where most of the energy is dissipated, is characterized by high-energy electrons; a successive spark phase, with a high density of low-energy electrons, presents favorable conditions for vibrational-mediated CO_2_ dissociation mechanisms.^29−31^ From 5 μs onward, we observe a decrease in the conversion. This finding can be rationalized by the combined effects of gas mixing and back-reactions reforming CO_2_.

Simulations performed by Heijkers et al.^32^ on a similar nanosecond pulsed discharge^23^ attributed the decrement of CO_2_ conversion mainly to the three-body recombination of carbon monoxide with atomic oxygen:
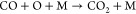
6where M represents a molecular colliding partner. Given its exothermic behavior, the back-reaction in eq 6 contributes to about 35% of gas heating; the other major contributor to the increment of the gas temperature is the vibration-to-translation channel.^32^ The theoretical predictions agree with the lag of the measured gas temperature w.r.t. the dissipation of the energy, as shown in Figure 2. Furthermore, the time evolution of the dissociation rates has been recently modeled by Pietanza et al.^33^ Although the results refer to a different discharge, it turns out that at early times, the electron-impact dissociation rate is larger than the vibrational-induced dissociation rate. However, the latter may overcome the former during the discharge progression, eventually increasing the total dissociation rate. Intriguingly, these findings somehow are akin to our observations and deserve further investigation with the support of computational models.

**Figure 3:**
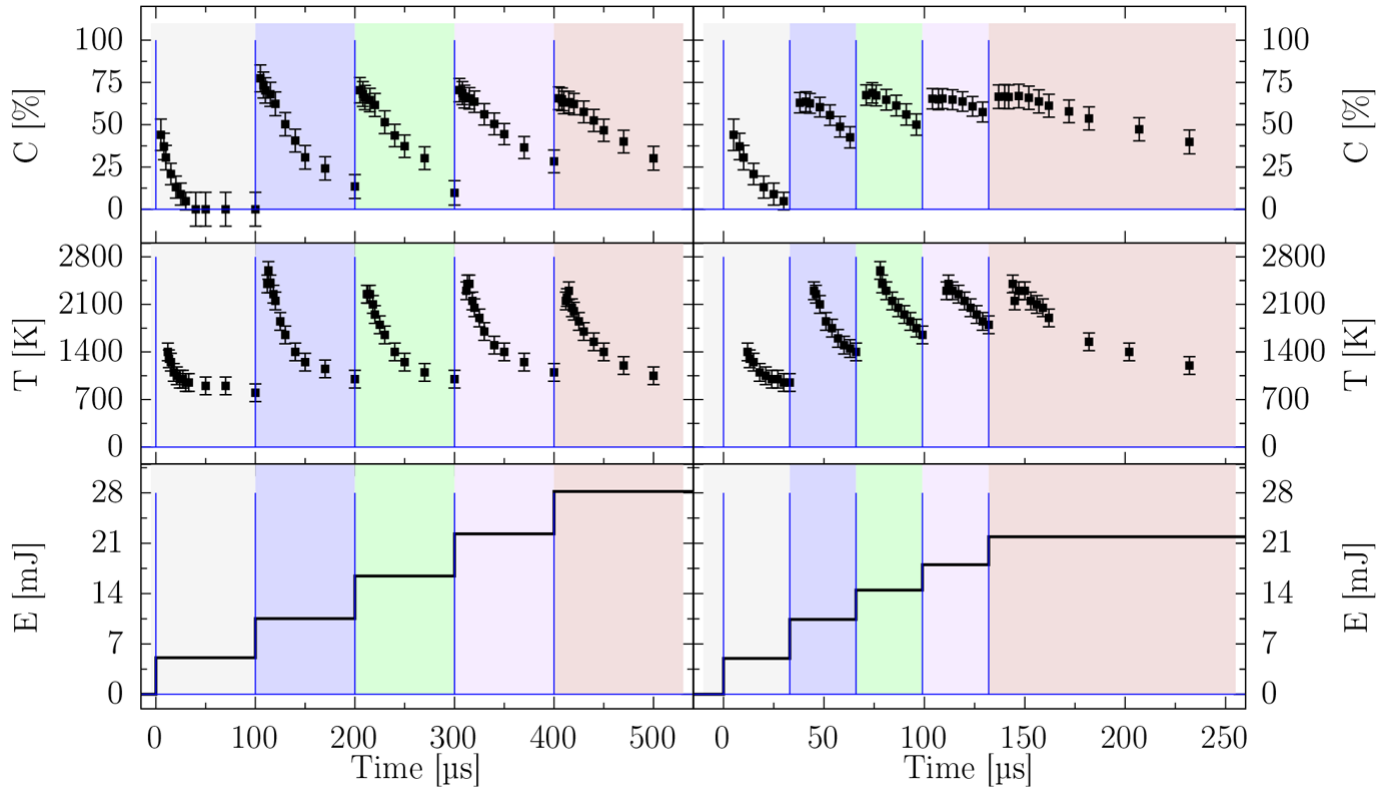
Top panel: CO_2_ conversion; mid panel: gas temperature; bottom panel: cumulative energy of the pulses inside the burst. Left: burst with *t*_p_ = 100 μs. Right: burst with *t*_p_ = 33 μs. The colored-shaded areas are a visual aid to distinguish subsequent pulses, along with the pulse train scheme (blue line).

**Figure 4:**
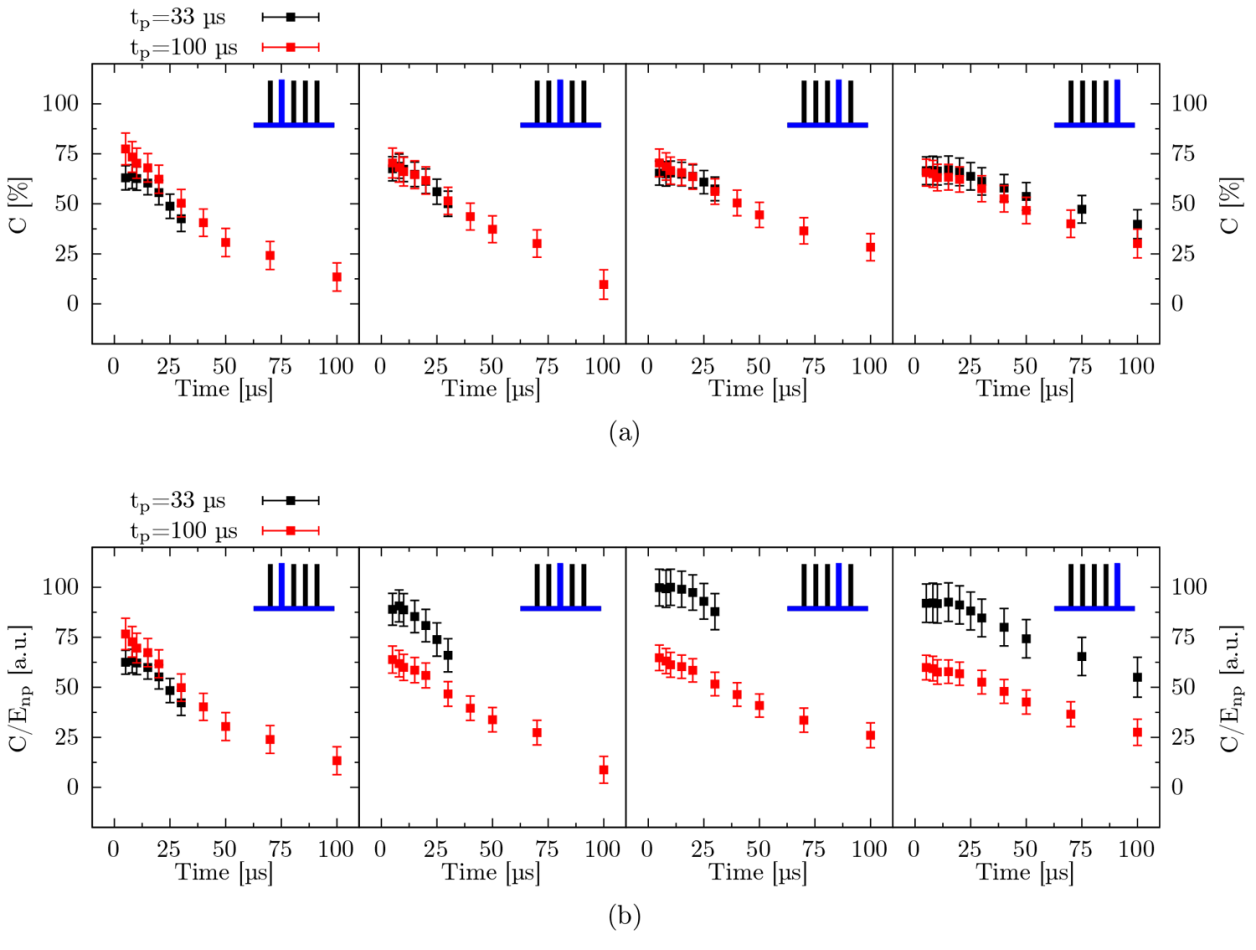
Comparison of the postdischarge conversion (a) and the pulse-energy-normalized conversion (b) for two burst sequences with different interpulse time (*t*_p_), for pulses *n* = 2–5. In the top right, there is a sketch of the 5-pulse burst with the corresponding pulse highlighted in blue. Initial pulses are not shown, since they would be equivalent.

Previous findings refer to a situation where the HV pulse regularly repeats with an interval long enough so that each pulse is independent of the previous one and, therefore, equivalent to it. Instead, the situation changes substantially when the interpulse time is short enough that a memory effect develops because successive discharges occur in a gas mixture perturbed by the preceding ones.^18,19,24^ We produced such a condition by using packets of five pulses, called bursts, where the temporal separation of the plasma pulses (*t*_p_; the interpulse time) could be varied; we chose *t*_p_ = 100 and 33 μs (see Figure 3). Typical values of conversion determined via gas chromatography at the exit of the reactor in similar operating conditions and with a specific energy input of 1.4 eV molecule^–1^ are 15 ± 1% for regularly repeated pulses and between 19 and 22% for burst with *t*_p_ < 100 μs.^19^ CO_2_ conversion *C* [%], gas temperature *T* [K], and cumulative energy of the bursts *E* [mJ] are presented in Figure 3. While *C* [%] and *T* [K] reach similar maximum values, the energy differs between the two interpulse time regimes. For *t*_p_ = 33 μs, the energy delivered to the plasma during the burst is 22 ± 1 mJ against the 28.0 ± 0.5 mJ for *t*_p_ = 100 μs. Since each pulse in a burst can have a different energy, we have divided the conversions by the energy of the respective *n*th pulse (*E*_np_). Figure 4 shows *C* and *C*/*E*_np_ from *n* = 2–5 pulse. While conversion values look similar, the pulse-energy-normalized conversion (*C*/*E*_p_) appear significantly different for 33 μs burst and the 100 μs burst.

In the former case, with smaller interpulse time, less energy (about 21%) has to be injected into the discharge to achieve similar conversion, indicating that energy is channeled more efficiently into CO_2_ dissociation. Therefore, by using short interpulse times, we avoid the breakdown phase and foster the spark phase, keeping the system in a quasi-“metastable” condition.

## Conclusions

We observed a delay of hundreds of nanoseconds between the discharge breakdown, the period when most of the energy is deposited, and when most of the CO_2_ dissociation occurs in a nanosecond repetitively pulsed plasma. In addition, by shortening the interpulse time, it is possible to increase the process efficiency. These findings indicate that the primary dissociation pathways are driven, and can be enhanced, by molecular-excitation kinetics, pointing to a role for vibrationally mediated processes.

## Supporting Information Available

Additional experimental details and methods on the electrical characterization, spectroscopic gas temperature, and CET-LIF (PDF)
