# Supplementary material for: CO2 Reduction by Nanosecond-Plasma Discharges: Revealing the Dissociation’s Time Scale and the Importance of Pulse Sequence
Source: J Phys Chem C Nanomater Interfaces. 2023 May 18;127(21):10045–50. doi: 10.1021/acs.jpcc.3c02547 (PMC10240531; doi:10.1021/acs.jpcc.3c02547)
Supplement: Supplementary file 1 — jp3c02547_si_001.pdf [file jp3c02547_si_001.pdf]

Supporting Information: CO_2 Reduction by Nanosecond-Plasma Discharges: Revealing the Dissociation's Time Scale and the Importance of Pulse Sequence

Cesare Montesano,[†] Toine P.W. Salden,[‡,†] Luca Matteo Martini,[*,†] Giorgio Dilecce,[¶,†] and Paolo Tosi[†,¶]

†*Department of Physics, University of Trento, Trento, 38123, Italy*

‡*Department of Applied Physics, Eindhoven University of Technology, Eindhoven, 5600 MB, Netherlands*

¶*CNR Institute for Plasma Science and Technology, Bari, 70126, Italy*

E-mail: luca.martini.1@unitn.it

Materials and Methods

Experimental set-up

The scheme of the experimental set-up is reported in Fig. S1, detailing the spectroscopic apparatus, as well as the gas system.

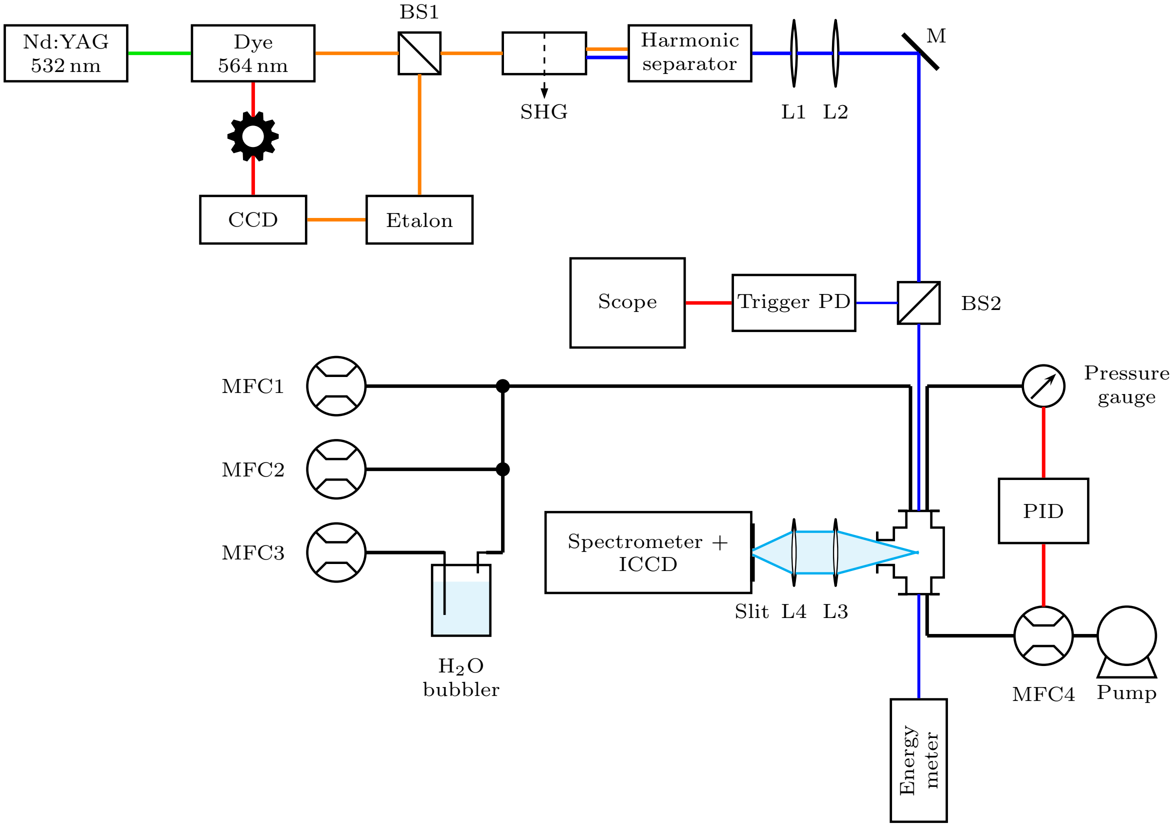

Fig. S1: In black: gas lines. In red: control signals. Green, orange and blue: laser beams. BS: beam splitters; SHG: second harmonic generator; L: lenses; M: mirrors; Trigger PD: photodiode used to trigger the oscilloscope (Scope); PID: proportional-integral-derivative controller; CCD+Etalon+Gear: feedback mechanism for the tuning of the dye.

Gas handling

The inlet gas flows were regulated by mass flow controllers MFC (1179A, MKS) MFC_1, MFC_2, MFC_3. The pressure in the reactor was stabilized by a proportional-integral-derivative (PID) controller acting on MFC_4 which was placed in the exhaust line. In the CET-LIF

experiment, water must be admixed to the CO_2 flow in order to produce OH. Specifically, 1% of H_2O was added by means of a temperature-stabilized bubbler. Adding only a small percentage of H_2O to the CO_2 stream implies that the fluorescence spectrum is not too sensive to slight variations of the H_2O concentration, see paragraph "CET-LIF".

Light detection

The detection system adopted for the optical emission spectroscopy and the collisional energy transfer laser-induced fluorescence (CET-LIF) consisted of a spectrograph equipped with a 300 mm focal length monochromator (SR303i-B, Shamrock, 2400, and 1200 grooves/mm gratings) and a gated ICCD (intensified charge-coupled device) camera (DH334T-18U-03, Andor iStar).

The light was collected by a lens L4 of focal length f_{L4}=200 mm placed at f_{L4} from the centre of the gap. A lens L3 with f_{L3}=75 mm served to focus the image on the entrance slit of the spectrograph.

Electrical characterization

The pulsing scheme adopted in the present work is presented in Fig. S2. A continuous pulse sequence with inter-pulse time t_c=5 ms was superimposed to a burst sequence characterized by an inter-burst time t_b=50 ms. The temporal separation of the nanosecond pulses inside the burst is indicated as t_p, the inter-pulse time t_p. The burst sequences employed in the present work consisted of five pulses and were characterized by t_p=100 µs and t_p=33 µs. The adoption of this modulated pulsing pattern reduced the total power delivered by the NPG18/100k compared to the burst sequences commonly used in plasma-mediated processes, also providing a better stability of the whole control system of the apparatus.

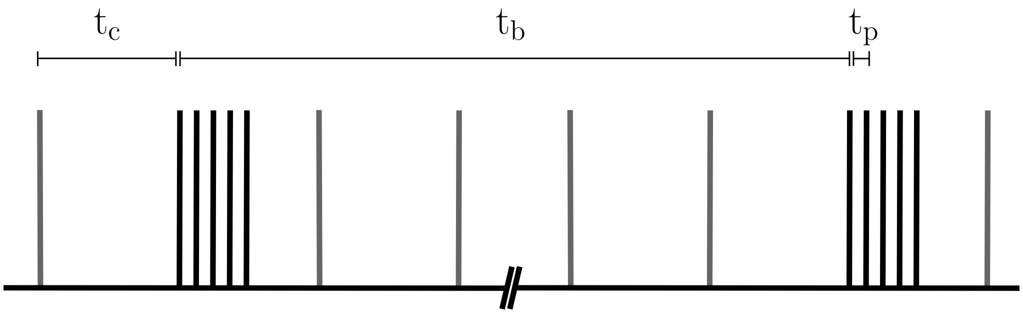

Fig. S2: The pulsing pattern composed of: a continuous pulse sequence with inter-pulse time t_c=5 ms, a superimposed burst sequence with inter-burst time t_b=50 ms. t_p is the inter-pulse time inside the burst. In this work, measurements were only performed in or after the burst.

Data analysis

The energy of the pulses was calculated by integrating the product V(t) x I(t) over the pulse duration, where V(t) and I(t) are the voltage and current signals, respectively. V(t) and I(t) were affected by a spurious delay introduced in the acquisition system for several reasons: length of the cables, separation of the probes, and the matching box of the high voltage probe. The delay was measured by preventing the discharge ignition and overlapping V(t) x I(t) so that the integral of the reactive power was zero. An example of voltage and current

signals is reported in Fig. S4. Instantaneous power P(t) and cumulative energy E(t) are shown in the panel Fig. S5. In both Fig. S4 and Fig. S5 a re-ignition of the discharge at around 400 ns can be observed in the 1st pulse (re-trigger of the NPG18/100k).

The profile of the 1st pulse of the burst depends on the temporal separation from the preceding pulse. As observed in,[1] provided that the discharge event that preceded the burst is distant enough – 5 ms in the case under study – the electrical characteristics of the 1st pulse are the same for the two sequences (t_p=100 µs and t_p=33 µs). Despite the presence of a re-ignition, about 85% of the total energy of the pulse is dissipated by the first discharge event, as shown in Fig. S3. The total energy deposited by the two burst sequences is presented in

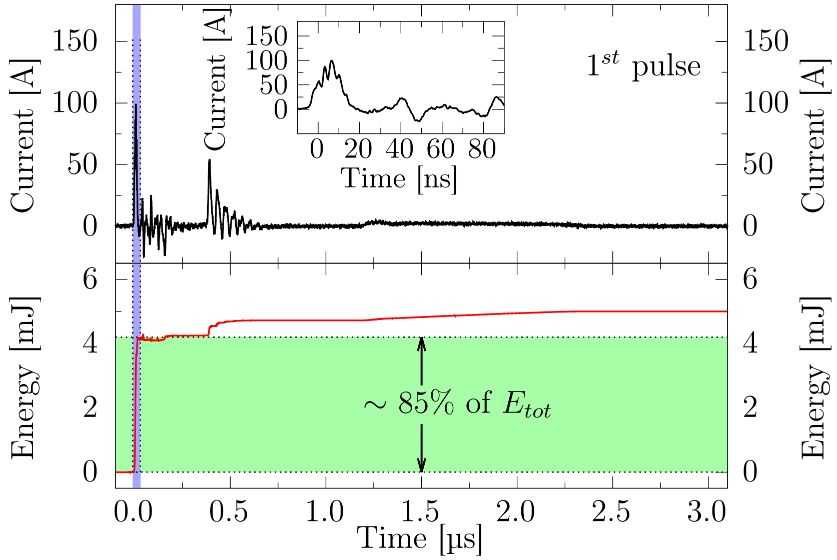

Fig. S3: Top panel: current of 1^{st}pulse of a burst with a macro of the first nanoseconds (indicated by shaded area). Bottom panel: cumulative energy.

Fig. S6. A remarkable difference in the total energy E_{tot} delivered by the two different burst sequences appeared. The burst with t_p=100 µs provided $E_{tot}^{100\,\mu s}$=28.2±0.4 mJ to the system; the burst with t_p=33 µs was characterized by a lower energy, specifically $E_{tot}^{33\,\mu s}$=22±1 mJ. Considering the uncertainty of E_{tot}, the discrepancy between $E_{tot}^{33\,\mu s}$ and $E_{tot}^{100\,\mu s}$ ranges from around 20% to 35%. Additionally, the burst sequences also differed by the profiles of voltage and current, as reported in the section "Additional voltage, current, power and energy profiles": Fig. S12-S13-S14-S15. This behaviour is consistent with what was observed in[1–3]

and with the so-called memory-dominated regime that appears by shortening t_p.

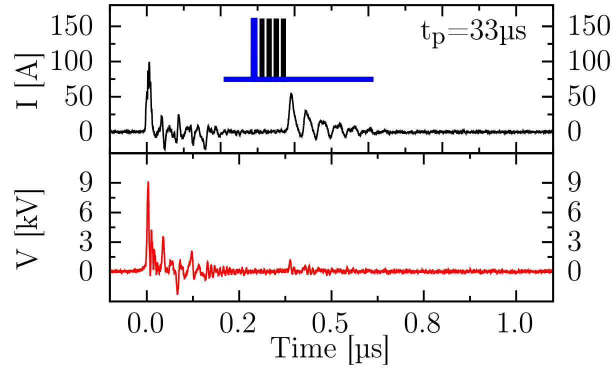

(a)

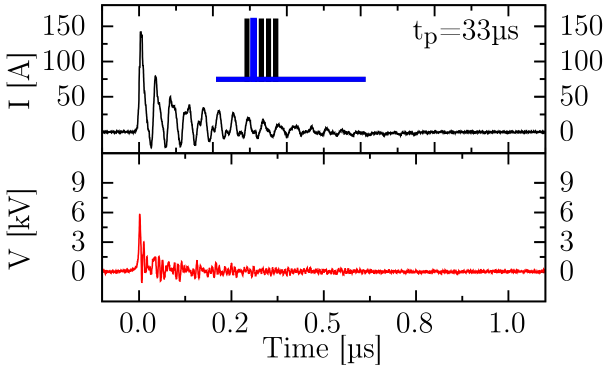

(b)

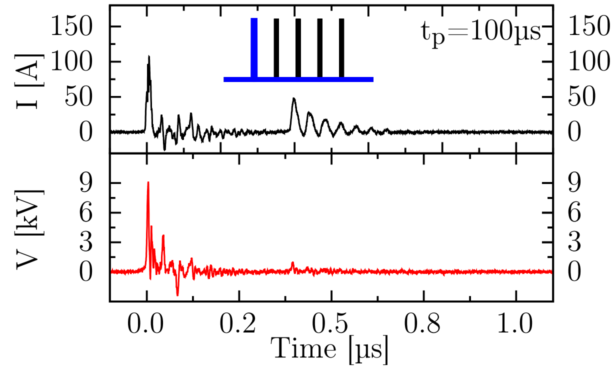

(c)

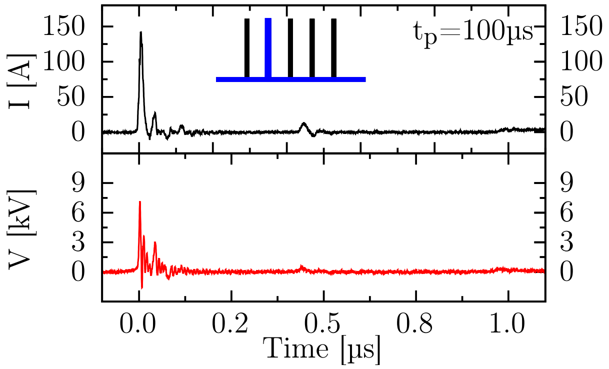

(d)

Fig. S4: Voltage V and current I profiles for the 33 µs (top row, a,b) and 100 µs (bottom row; c, d) conditions, for both the first (a,c) and second (b,d) pulses respectively.

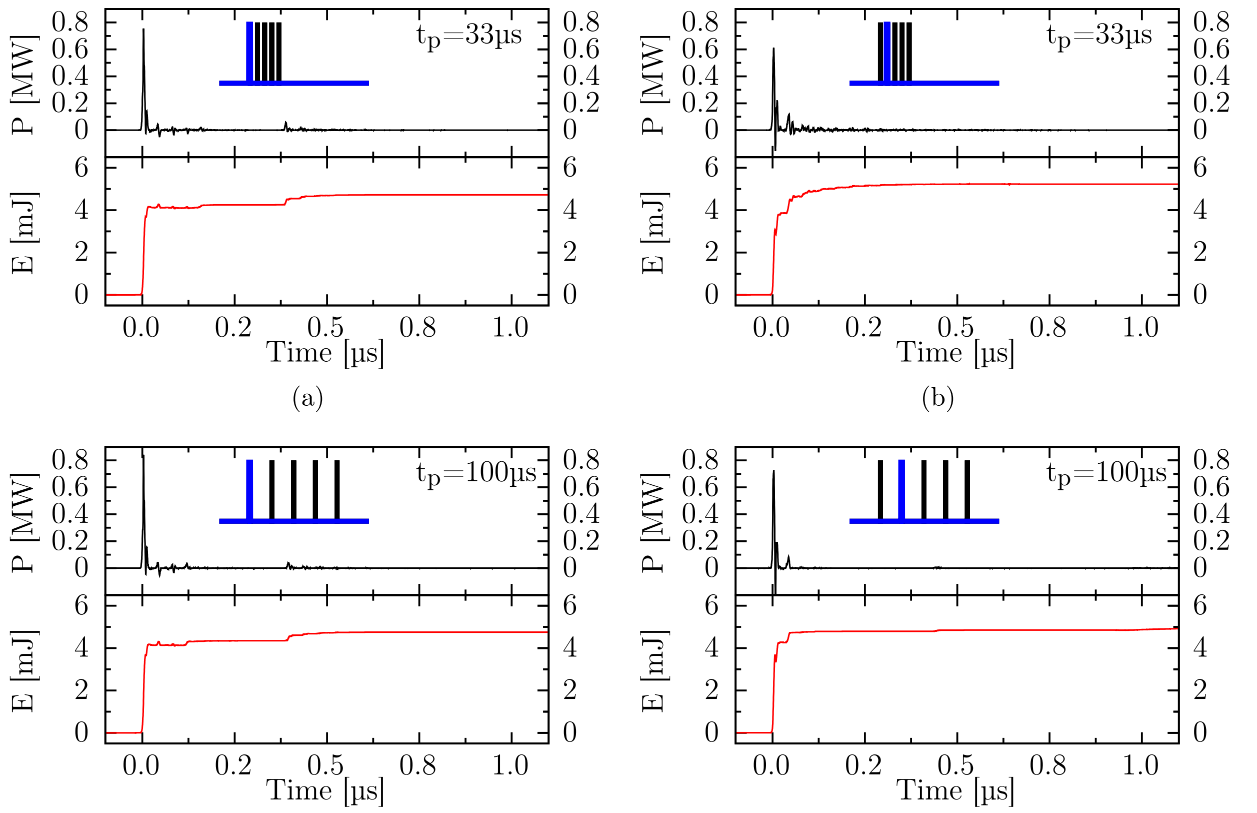

(a) (b)

(c) (d)

Fig. S5: Instantaneous power P and cumulative energy E for the 33 µs (top row, a,b) and 100 µs (bottom row; c, d) conditions, for both the first (a,c) and second (b,d) pulses respectively.

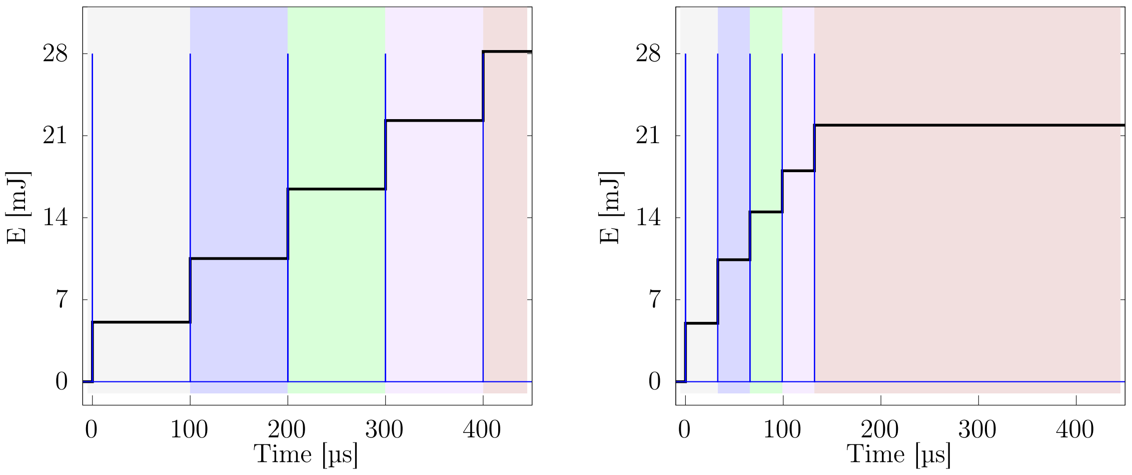

(a) (b)

Fig. S6: Cumulative energy of the pulses inside the burst. (a) Burst with t_p=100 µs. (b) Burst with t_p=33 µs. In blue is a pictorial representation of the nanosecond discharge pulses.

Spectroscopic gas temperature

The gas temperature can be estimated from optical emission spectra, specifically using the emission of the second positive system (SPS) of nitrogen,[4,5] in an atmospheric nanosecond pulsed discharge.[3] In this work, the $N_2(C^3\Pi_u,\, v' = 0)$ state was produced by the discharge itself and the signal was collected in the first 20 ns of the discharge pulse.

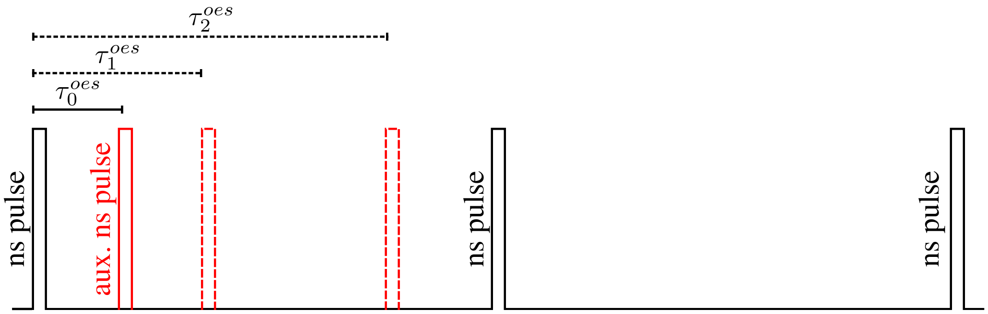

Fig. S7: Timing of the discharge pulses in an optical emission spectroscopy experiment for the determination of the gas temperature from emission of the Second Positive System of nitrogen. ns pulse represents the discharge event to be probed; the aux. ns pulses are the additional pulses used to produced the state $N_2(C^3\Pi_u,\, v' = 0)$ at a given delay τ^{oes} by the ns. pulse.

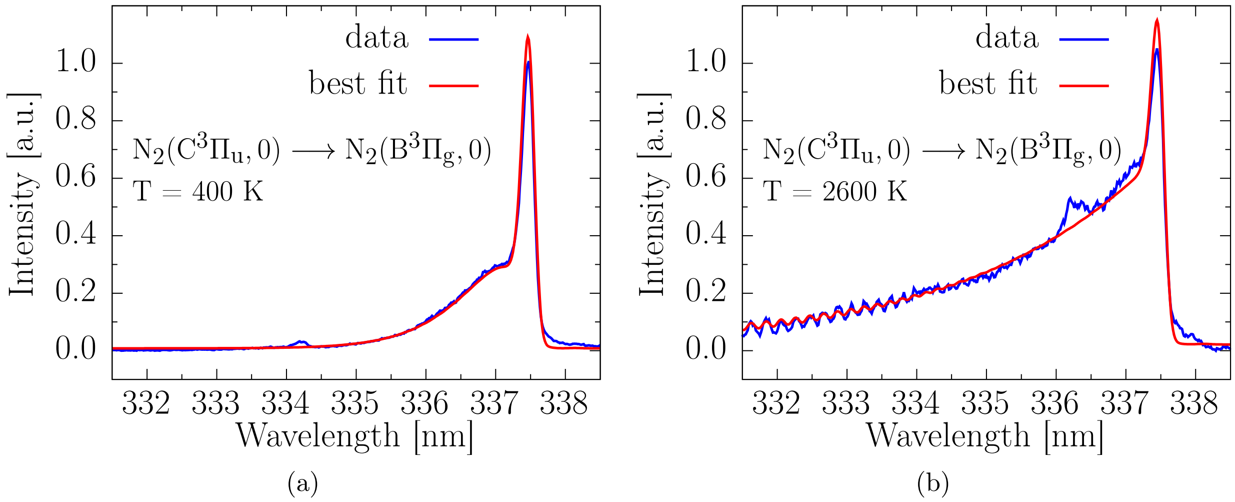

Fig. S8: Second positive system emission spectra representative of T_g=400 K (a) and T_g=2600 K (b). The synthetic spectra were simulated using the software DIATOMIC[6]

To span the post-discharge of each pulse of the burst sequence, an auxiliary nanosecond

(aux. ns. pulse) pulse was used to produce the state $N_2(C^3\Pi_u, \upsilon' = 0)$, see Fig. S7. The aux. ns. pulse was progressively delayed (τ_0^{oes}, τ_1^{oes}, τ_2^{oes}, ...) with respect to the ns. pulse to be probed by using the DDG. The auxiliary pulse cannot be closer than 10 µs with respect to the pulse of interest since it is the minimum achievable inter-pulse time for the NPG18/100k power supply. The sketch of the timing of the experiment is shown in Fig. S7.

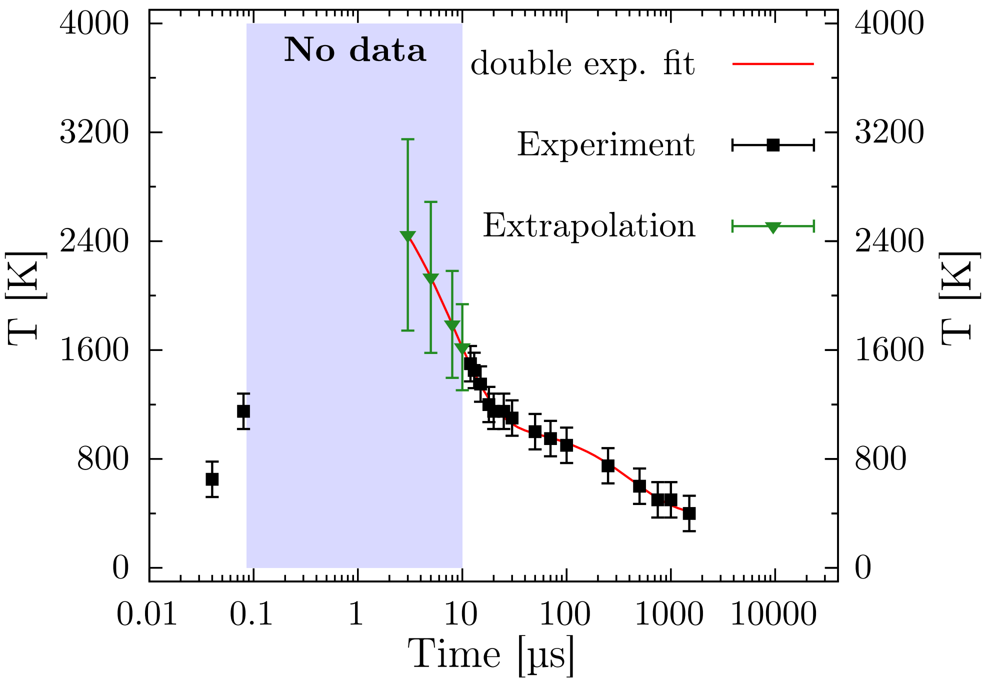

Fig. S9: Double exponential fit of the post-discharge gas temperature. The blue shaded area represents the non-accessible region because of the absence of electron impact excitation of $N_2(C^3\Pi_u, \upsilon' = 0)$.

Data analysis

Two examples of emission spectra produced by the second positive system of nitrogen $N_2(C^3\Pi_u, \upsilon' = 0) \rightarrow N_2(B^3\Pi_g, \upsilon = 0)$ are shown in Fig. S8. To find the best fit of the data, a set of synthetic spectra was produced by using the software DIATOMIC[6] in a temperature range of [280 K-4000 K], with a temperature step of dT=50 K. Each data-set was fitted on each synthetic spectrum, the best fit corresponds to the minimum value of the χ^2_{red}.

The uncertainty was derived by using the χ^2_{red} procedure, as presented in.[7] The data are fitted on a much more granular set of synthetic spectra (dT=5 K); the resulting dependence of the χ^2_{red} with respect to T [K] is a parabola with half width $\chi^2_{\text{red,min}} + 0.5$, representing the uncertainty; it is 130 K for the measurements presented in this work.

Additionally, if a fit is performed on the post-discharge evolution of the gas temperature, all the uncertainties on the fit parameters must be considered in the extrapolation of the temperature values. An example of the extrapolation in the non-accessible region by the auxiliary pulse is presented in Fig. S9. The experimental point at 40 ns and 80 ns were collected because the discharge was ignited by the first reflection travelling back and fourth in the cable, see Fig. S3.

CET-LIF

Collisional energy transfer - laser induced fluorescence (CET-LIF) was introduced in[8] to derive the gas composition in a highly collisional environment, e.g. atmospheric pressure discharges. The collisional processes undergone by the probe molecule – OH in the specific case – affect its fluorescence spectrum. Despite that operation in a highly collisional environment is detrimental for the LIF quantum yield,[9] if the species-dependent rate coefficients of those processes are known, the information on the gas composition can be inferred by the fluorescence spectrum of OH. The set of non-thermal rate coefficient $k(T)_{Q1}$, $k(T)_{Q0}$, $k(T)_{1\rightarrow 0}$[a] for several collider partners of OH (CO_2, CO, O_2, H_2, CH_4) was measured in.[10] The temperature dependence of the $k(T)$ was also highlighted.

The spectroscopic scheme used in this work corresponds to the one adopted in.[11,12] The notation is the following: the vibrational (υ) and rotational (N) quantum numbers of the states $\mathrm{OH}\big(\mathrm{X}^2\Pi, \upsilon, \mathrm{N}'\big)$ and $\mathrm{OH}\big(\mathrm{A}^2\Sigma^+, \upsilon', \mathrm{N}''\big)$ will be indicated with their integer values. If the rotational quantum number is omitted, the state represents the rotational manifold. The spectroscopic scheme was as follows:

$$\begin{aligned} \mathrm{OH}\big(\mathrm{X}^2\Pi, \upsilon = 0\big) + h\nu_\mathrm{L} \longrightarrow & \mathrm{OH}\big(\mathrm{A}^2\Sigma^+, \upsilon' = 1\big) \longrightarrow \mathrm{OH}\big(\mathrm{X}^2\Pi, \upsilon = 1\big) + \mathrm{h}\nu_{(1,1)} \\ & \mathrm{VET}\Big\downarrow \\ & \mathrm{OH}\big(\mathrm{A}^2\Sigma^+, \upsilon = 0\big) \longrightarrow \mathrm{OH}\big(\mathrm{X}^2\Pi, \upsilon = 0\big) + \mathrm{h}\nu_{(0,0)}. \end{aligned} \tag{S1}$$

The $\mathrm{P}_1(3)$ transition at 283.009 nm was chosen to excite the $\mathrm{OH}\big(\mathrm{A}^2\Sigma^+, \upsilon' = 1\big)$ state. The laser-populated state could undergo different energy transfer processes: i) rotational energy-transfer (RET) in which the energy is redistributed in the rotational manifold; ii) vibrational energy-transfer in which $\mathrm{OH}\big(\mathrm{A}^2\Sigma^+, \upsilon' = 1\big)$ is depopulated in favour of $\mathrm{OH}\big(\mathrm{A}^2\Sigma^+, \upsilon' = 0\big)$; iii) electronic quenching: non-radiative decay of the $\mathrm{OH}\big(\mathrm{A}^2\Sigma^+, \upsilon' = 1\big)$ state. The time-

[a] $k(T)_{Q1}$, $k(T)_{Q0}$, $k(T)_{1\rightarrow 0}$ are the rate coefficients corresponding to the quenching of $\mathrm{OH}\big(\mathrm{A}^2\Sigma^+, \upsilon' = 1\big)$, $\mathrm{OH}\big(\mathrm{A}^2\Sigma^+, \upsilon' = 0\big)$ and the vibrational energy-transfer $\mathrm{OH}\big(\mathrm{A}^2\Sigma^+, \upsilon' = 1\big) \rightarrow \mathrm{OH}\big(\mathrm{A}^2\Sigma^+, \upsilon' = 0\big)$

integrated fluorescence spectrum $I(\lambda)_{\mathrm{LIF}}$ was acquired by the intensified CCD. $I(\lambda)_{\mathrm{LIF}}$ can be expressed as the sum of the contributions coming from the two emitting bands (0,0) and (1,1) (see the spectroscopic scheme of Eq. S1):

$$\mathrm{I}(\lambda)_{\mathrm{LIF}} = \mathrm{I}(\lambda)_0 + \mathrm{I}(\lambda)_1 \propto \psi_{(0,0)}(\lambda) \int_0^{\mathrm{t_G}} \mathrm{P}_0(\mathrm{t})\mathrm{dt} + \psi_{(1,1)}(\lambda) \int_0^{\mathrm{t_G}} \mathrm{P}_1(\mathrm{t})\mathrm{dt} \tag{S2}$$

$$\mathrm{I}(\lambda)_0 : \quad \mathrm{OH}\left(\mathrm{A}^2\Sigma^+, v' = 0\right) \longrightarrow \mathrm{OH}\left(\mathrm{X}^2\Pi, v = 0\right), \ (0,0) \text{ band}$$

$$\mathrm{I}(\lambda)_1 : \quad \mathrm{OH}\left(\mathrm{A}^2\Sigma^+, v' = 1\right) \longrightarrow \mathrm{OH}\left(\mathrm{X}^2\Pi, v = 1\right), \ (1,1) \text{ band.}$$

$\psi(\lambda)_{(0,0)}$ and $\psi(\lambda)_{(1,1)}$ are the normalized emission spectra of the band (0,0) and (1,1) for a given rotational population distribution, respectively. $\mathrm{t_G}$ is the gate-time of the ICCD, fixed to 20 ns to be larger than the lifetime of the laser-populated state. $\mathrm{P}_0(\mathrm{t})$ and $\mathrm{P}_1(\mathrm{t})$ are the time-dependent vibrational populations. The ratio ($\mathrm{R_p}$) between the time-integrated vibrational populations can be expressed as the ratio between the effective rate coefficients of VET ($k^{\mathrm{eff}}_{\mathrm{VET}}$) and quenching ($k^{\mathrm{eff}}_{\mathrm{Q0}}$) of the state $\mathrm{OH}\left(\mathrm{A}^2\Sigma^+, v' = 1\right)$:[13]

$$\mathrm{R_p} = \frac{\int_0^{\mathrm{t_G}} \mathrm{P}_0(\mathrm{t})\mathrm{dt}}{\int_0^{\mathrm{t_G}} \mathrm{P}_1(\mathrm{t})\mathrm{dt}} = \frac{\mathrm{k}^{\mathrm{eff}}_{\mathrm{VET}}}{\mathrm{k}^{\mathrm{eff}}_{\mathrm{Q0}}}$$
$$= \frac{\left((1-\gamma)\cdot \mathrm{k}^{\mathrm{CO_2}}_{\mathrm{VET}} + \gamma \cdot \mathrm{k}^{\mathrm{CO}}_{\mathrm{VET}} + \frac{\gamma}{2}\cdot \mathrm{k}^{\mathrm{O_2}}_{\mathrm{VET}}\right)\cdot \left[\mathrm{CO_2}\right]_{\mathrm{in}} + \mathrm{k}^{\mathrm{H_2O}}_{\mathrm{VET}} \cdot \left[\mathrm{H_2O}\right]_{\mathrm{in}}}{\left((1-\gamma)\cdot \mathrm{k}^{\mathrm{CO_2}}_{\mathrm{Q_0}} + \gamma \cdot \mathrm{k}^{\mathrm{CO}}_{\mathrm{Q_0}} + \frac{\gamma}{2}\cdot \mathrm{k}^{\mathrm{O_2}}_{\mathrm{Q_0}}\right)\cdot \left[\mathrm{CO_2}\right]_{\mathrm{in}} + \mathrm{k}^{\mathrm{H_2O}}_{\mathrm{Q_0}} \cdot \left[\mathrm{H_2O}\right]_{\mathrm{in}}} \tag{S3}$$

Where $[\mathrm{CO_2}]_{\mathrm{in}}$, $[\mathrm{H_2O}]_{\mathrm{in}}$ are the initial molar fractions of $\mathrm{CO_2}$ and $\mathrm{H_2O}$, respectively. γ can be derived by Eq. S3 if $\mathrm{R_p}$ is known. To derive γ along the nanosecond pulse sequences, the laser pulse is progressively delayed by τ^{lif} with respect to the nanosecond pulse, see Fig. S10. In the next paragraph the procedure to derive the time-integrated vibrational population ratio is detailed. To simplify the visualization of the graphs, the $\mathrm{CO_2}$ dissociation γ (multiplied by 100) will be indicated as the conversion percentage C [%].

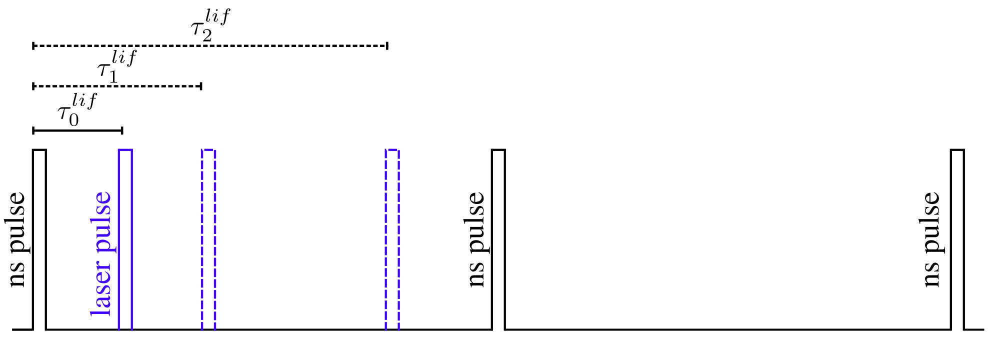

Fig. S10: Timing of the discharge pulses in a CET-LIF experiment for the determination of the CO_2 conversion at microsecond timescales. The label 'ns pulse' represents the discharge event to be probed; the label 'laser pulse' represents the probing laser pulse that produces the excited state $\mathrm{OH}\big(\mathrm{A}^2\Sigma^+,\, \upsilon' = 1\big)$ at a given delay τ^{lif} by the ns pulse.

Data analysis

The spectral fitting routine used in [10] was adopted to extract the rotational population distributions of $\mathrm{OH}\big(\mathrm{A}^2\Sigma^+,\, \upsilon' = 0\big)$ and $\mathrm{OH}\big(\mathrm{A}^2\Sigma^+,\, \upsilon' = 1\big)$, together with the ratio described in Eq. S3. If the rotational population distributions of the investigated state of OH are known, the fitting routine can be simplified by reducing the number of fitting parameters. In this framework, the number of rotational levels of the manifolds $\upsilon = 1$ and $\upsilon = 0$ was fixed and the fitting function for $I(\lambda)_{\mathrm{LIF}}$ that can be parameterized as a linear combination of the normalized emission spectra, see Eq. S4:

$$\mathrm{I}(\lambda)_{\mathrm{LIF}} = \mathrm{a} \cdot \psi(\lambda)_{(0,0)} + \mathrm{b} \cdot \psi(\lambda)_{(1,1)} = \mathrm{I}(\lambda)_0 + \mathrm{I}(\lambda)_1 \tag{S4}$$

$$\frac{\mathrm{a}}{\mathrm{b}} = \frac{\int_0^{\mathrm{t_G}} \mathrm{P}_0(\mathrm{t})\mathrm{dt}}{\int_0^{\mathrm{t_G}} \mathrm{P}_1(\mathrm{t})\mathrm{dt}} \tag{S5}$$

where a and b are the fitting parameters. To validate this approach, the rotational population distributions were probed at different $\tau^{\mathrm{lif}}_{\mathrm{rot}}$ and the data-sets collected at $\tau^{\mathrm{lif}} \neq \tau^{\mathrm{lif}}_{\mathrm{rot}}$ were fitted using the simplified fitting function of Eq. S4[b]. An example of the procedure is provided in Fig. S11. The first two rows show three different fits for a fluorescence spectrum acquired at a

[b]the fitting routing mentioned above was used to fit resolved spectra acquired at specific τ^{lif}.

given τ^{lif} (30 µs and 5 µs). Each fit is performed by using a rotational population distribution measured at $\tau_{\mathrm{rot}}^{\mathrm{lif}}$ =1, 5, 30 µs, respectively. The values of the ratios are compatible for the different $\tau_{\mathrm{rot}}^{\mathrm{lif}}$, implying that the rotational population distributions do not vary in a sensitive way to affect $\mathrm{R_p}$. The rotational population distributions are presented in the third row.

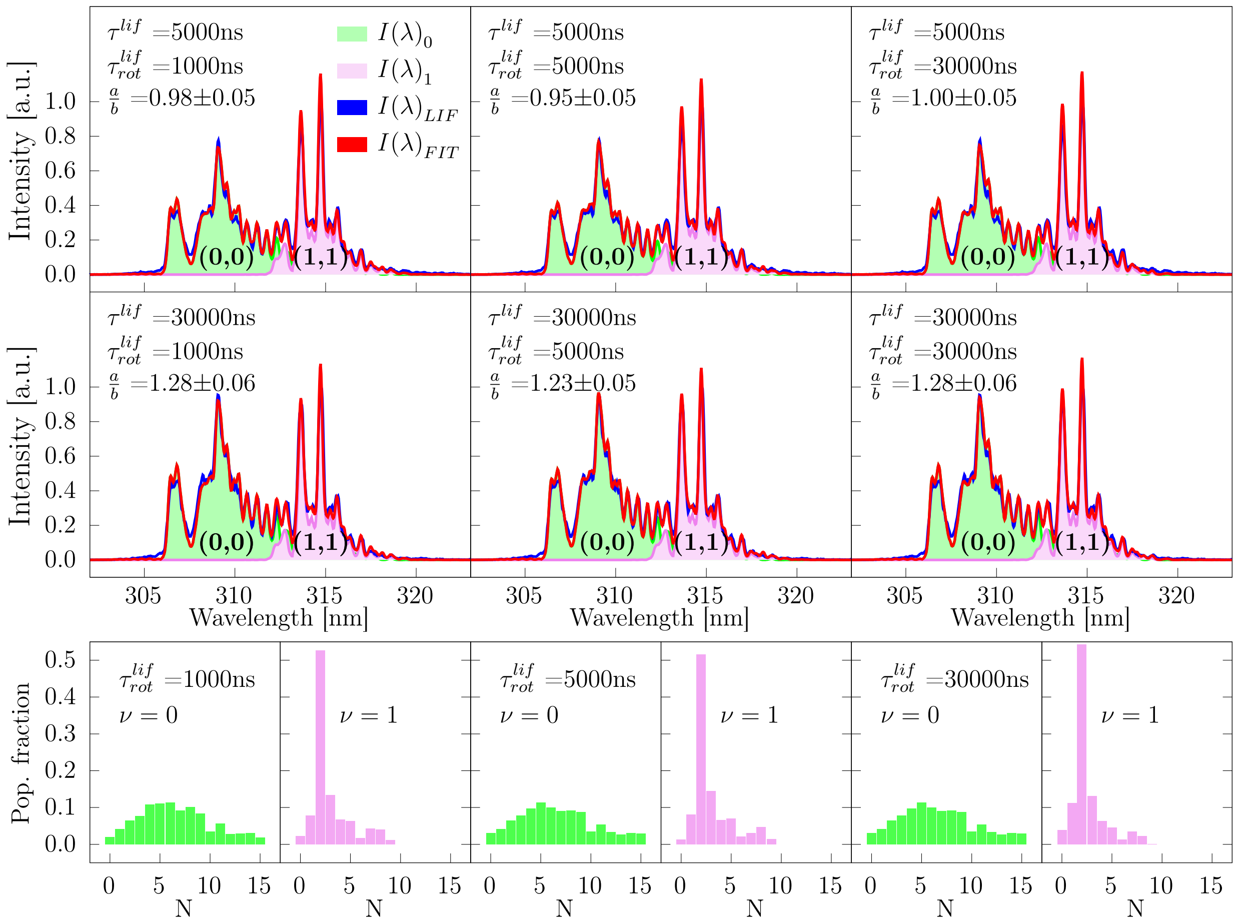

Fig. S11: **First row**: data-set acquired at τ^{lif}=5 µs and fitted over the rotational population distributions at $\tau_{\mathrm{rot}}^{\mathrm{lif}}$ =1, 5, 30 µs, respectively. **Second row**: data-set acquired at τ^{lif}=30 µs and fitted over the rotational population distribution at $\tau_{\mathrm{rot}}^{\mathrm{lif}}$ =1, 5, 30 µs, respectively. **Third row**: rotational population distributions at $\tau_{\mathrm{rot}}^{\mathrm{lif}}$ =1, 5, 30 µs, respectively. The outlier in the population fractions of the $\mathrm{OH}\left(\mathrm{A}^2\Sigma^+,\, v'=1\right)$ state corresponds to the laser populated state.

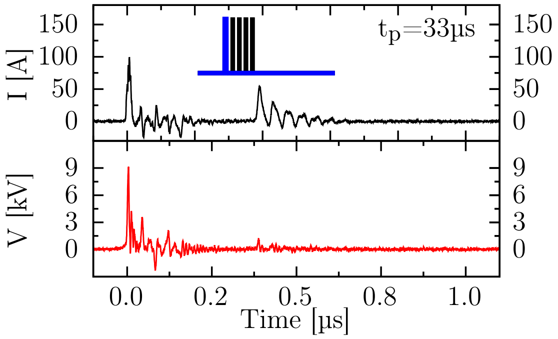

(a)

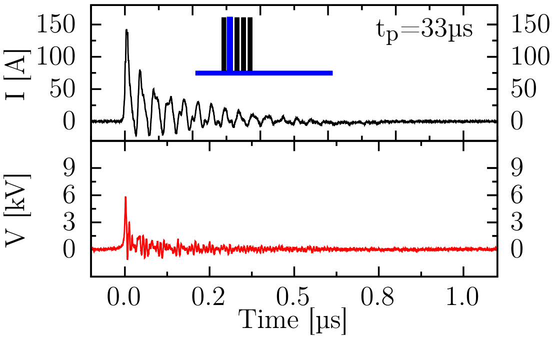

(b)

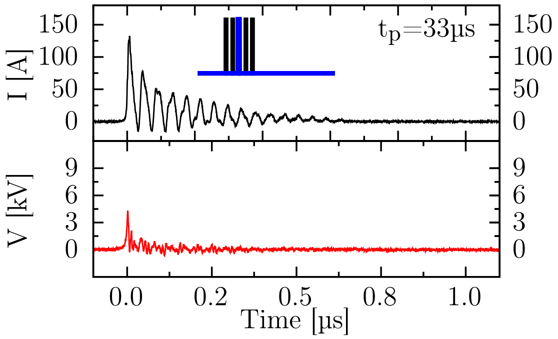

(c)

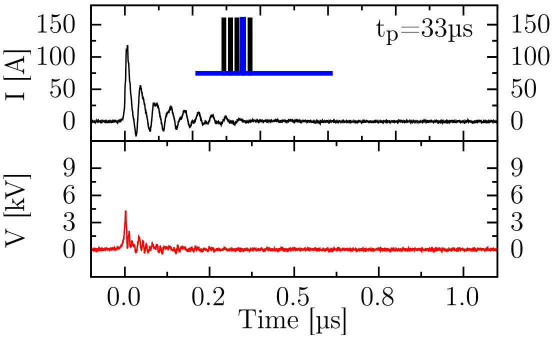

(d)

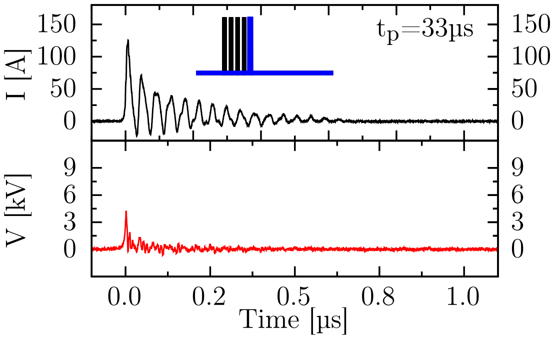

(e)

Fig. S12: Voltage V and current I profiles for the five pulses of the burst with t_p=33 µs. Subfigures (a) to (e) correspond to pulse indices 1 to 5 respectively, as can also be seen from the blue highlighted pulse in the pictrographic burst representation in each graph.

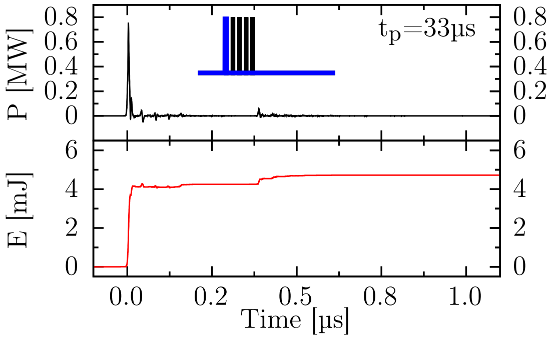

(a)

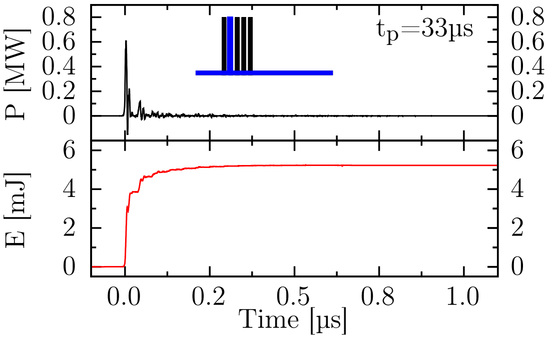

(b)

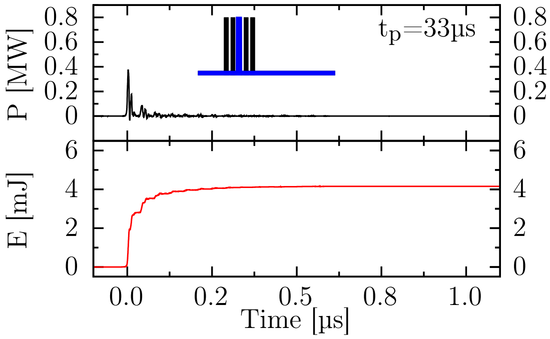

(c)

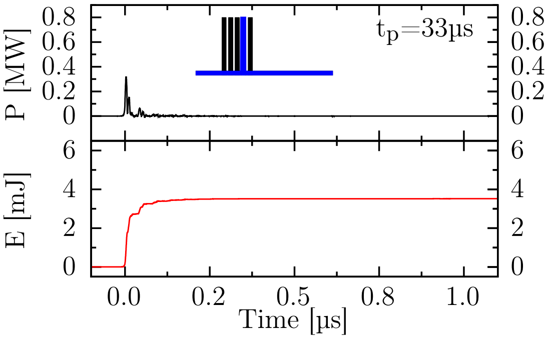

(d)

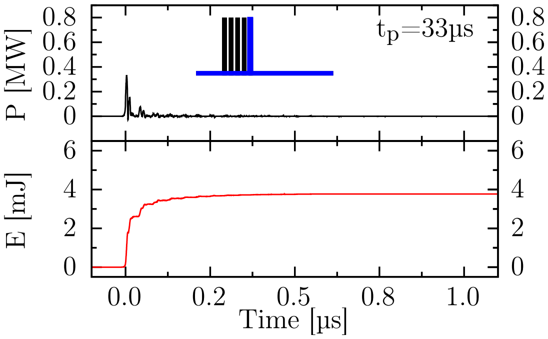

(e)

Fig. S13: Instantaneous power P and energy E profiles for the five pulses of the burst with t_p=33 µs. Subfigures (a) to (e) correspond to pulse indices 1 to 5 respectively, as can also be seen from the blue highlighted pulse in the pictrographic burst representation in each graph.

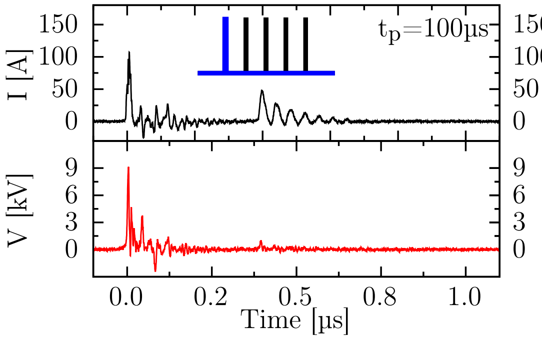

(a)

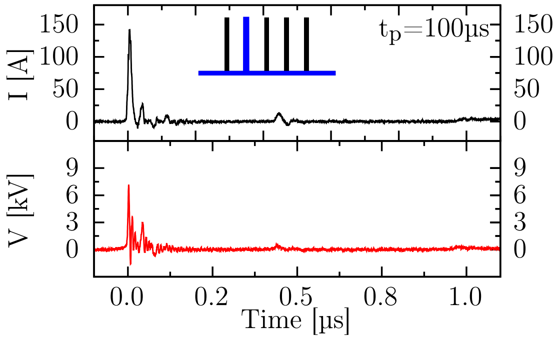

(b)

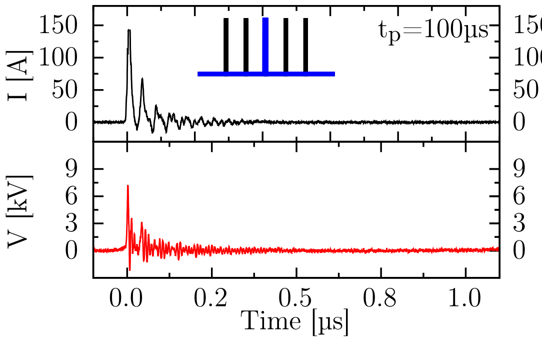

(c)

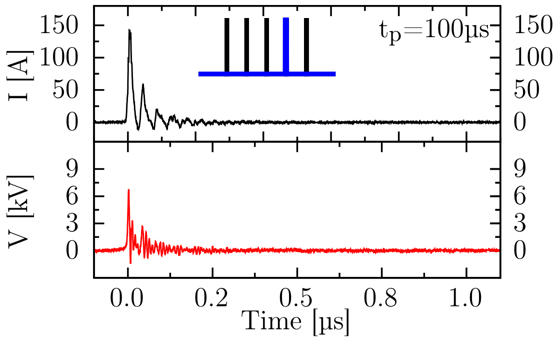

(d)

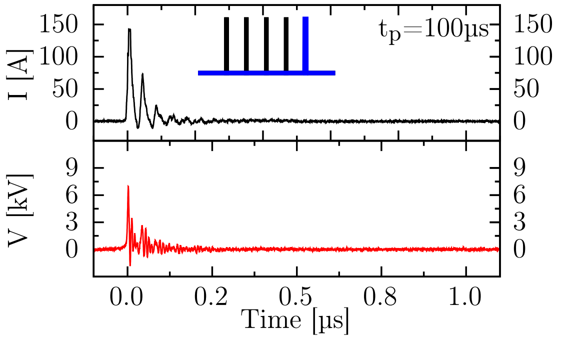

(e)

Fig. S14: Voltage V and current I profiles for the five pulses of the burst with t_p=100 µs. Subfigures (a) to (e) correspond to pulse indices 1 to 5 respectively, as can also be seen from the blue highlighted pulse in the pictrographic burst representation in each graph.

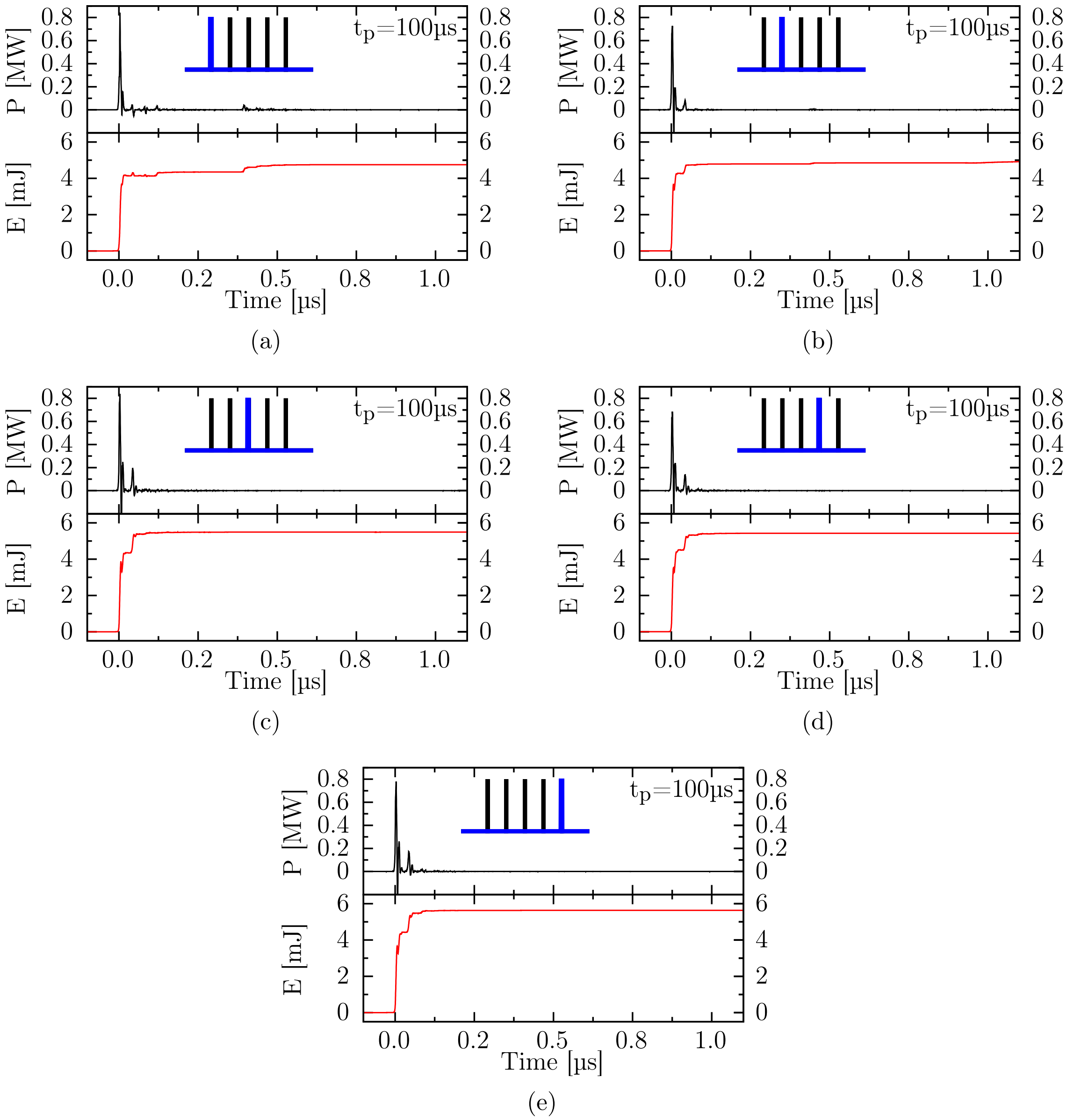

Fig. S15: Instantaneous power P and energy E profiles for the five pulses of the burst with t_p=100 µs. Subfigures (a) to (e) correspond to pulse indices 1 to 5 respectively, as can also be seen from the blue highlighted pulse in the pictrographic burst representation in each graph.

References

(1) Montesano, C.; Quercetti, S.; Martini, L. M.; Dilecce, G.; Tosi, P. The effect of different pulse patterns on the plasma reduction of CO_2 for a nanosecond discharge. *J. CO_2 Utiliz.* **2020**, *39*, 101157.

(2) Montesano, C.; Faedda, M.; Martini, L. M.; Dilecce, G.; Tosi, P. CH_4 reforming with CO_2 in a nanosecond pulsed discharge. The importance of the pulse sequence. *J. CO_2 Utiliz.* **2021**, *49*, 101556.

(3) Ceppelli, M.; Salden, T. P. W.; Martini, L. M.; Dilecce, G.; Tosi, P. Time-resolved optical emission spectroscopy in CO_2 nanosecond pulsed discharges. *Plasma Sources Sci. Technol.* **2021**, *30*, 115010.

(4) Bruggeman, P. J.; Sadeghi, N.; Schram, D. C.; Linss, V. Gas temperature determination from rotational lines in non–equilibrium plasmas: a review. *Plasma Sources Sci. Technol.* **2014**, *23*, 023001.

(5) Laux, C. O.; Spence, T. G.; Kruger, C. H.; Zare, R. N. Optical diagnostics of atmospheric pressure air plasmas. *Plasma Sources Sci. Technol.* **2003**, *12*, 125–138.

(6) Tan, X. *Diatomic, a spectral simulation program for diatomic molecules on windows platforms*; 2006.

(7) Klarenaar, B. L. M.; Engeln, R.; van den Bekerom, D. C. M.; van de Sanden, M. C. M.; Morillo-Candas, A. S.; Guaitella, O. Time evolution of vibrational temperatures in a CO_2 glow discharge measured with infrared absorption spectroscopy. *Plasma Sources Sci. Technol.* **2017**, *26*, 115008.

(8) Martini, L. M.; Gatti, N.; Dilecce, G.; Scotoni, M.; Tosi, P. Laser induced fluorescence in nanosecond repetitively pulsed discharges for CO_2 conversion. *Plasma Phys. Control. Fusion* **2018**, *60*, 014016.

(9) Dilecce, G. Optical spectroscopy diagnostics of discharges at atmospheric pressure. *Plasma Sources Sci. Technol.* **2014**, *23*, 015011.

(10) Ceppelli, M.; Martini, L. M.; Dilecce, G.; Scotoni, M.; Tosi, P. Non-thermal rate constants of quenching and vibrational relaxation in the OH $(A^2\Sigma^+, \nu' = 0, 1)$ manifold. *Plasma Sources Sci. Technol.* **2020**, *29*, 065019.

(11) Dilecce, G.; Martini, L. M.; Tosi, P.; Scotoni, M.; Benedictis, S. D. Laser induced fluorescence in atmospheric pressure discharges. *Plasma Sources Sci. Technol.* **2015**, *24*, 034007.

(12) Martini, L. M.; Lovascio, S.; Dilecce, G.; Tosi, P. Time-Resolved CO_2 Dissociation in a Nanosecond Pulsed Discharge. *Plasma Chem. Plasma Process.* **2018**, *38*, 707–718.

(13) Dilecce, G.; Martini, L. M.; Ceppelli, M.; Scotoni, M.; Tosi, P. Progress on laser induced fluorescence in a collisional environment: the case of OH molecules in ns pulsed discharges. *Plasma Sources Sci. Technol.* **2019**, *28*, 025012.
